# Development and validation of the Breast Cancer Myths Scale for women

**DOI:** 10.1590/1806-9282.20241627

**Published:** 2025-06-02

**Authors:** Maide Nur Tümkaya, Şehma Şen, Kafiye Eroğlu

**Affiliations:** 1 Koç University, School of Nursing – İstanbul, Turkey.; 2 Istanbul Atlas University, Faculty of Health Sciences – İstanbul, Turkey.

**Keywords:** Breast cancer, Belief, Scale, Women

## Abstract

**OBJECTIVE:**

The aim of this study was to develop and validate the Breast Cancer Myths Scale for women.

**METHODS:**

Confirmatory and exploratory factor analyses were used in this methodological study. The internal consistency was evaluated using the item–total correlation.

**RESULTS:**

The root mean square error of approximation was calculated as 0.072. The final scale version demonstrated excellent internal consistency (Cronbach's alpha=0.908).

**CONCLUSION:**

The Breast Cancer Myths Scale (single factor, 16 items) is a reliable and valid measure that can be used to evaluate breast cancer myths.

## INTRODUCTION

Despite advances in medical science and increased awareness, numerous myths and misconceptions about breast cancer (BC) persist within society. Misconceptions and myths about BC, such as believing that only women with a family history of the disease are at risk, can lead to complacency and delayed detection. Dispelling these myths and providing factual information is essential for encouraging early detection and proactive prevention strategies, which can significantly improve health outcomes^1,2^.

Currently, there is no scientific measurement tool available to identify the myths about BC that are commonly believed. This gap has led to a lack of scientific data on these myths, posing a significant obstacle to understanding their prevalence and impact. By identifying and measuring these misconceptions, we can design educational programs and public health campaigns that effectively counteract misinformation and promote factual knowledge. This study aimed to develop a measurement tool specifically for identifying BC myths in society.

## METHODS

A multi-step approach, which includes item generation, scale development, and scale validation, was utilized in this methodological research. In phase 1, the initial item pool was developed based on an extensive literature review. In phase 2, an expert committee review was conducted to evaluate the importance of the items. A pretest and interview were then conducted among a small sample of 12 women from different sociocultural levels. The participants completed the scale, explained their own beliefs about BC, and provided feedback on the scale's applicability through a focus group interview. In phase 3, a face-to-face questionnaire was used to gather information from participants at three different family health centers in Türkiye. After the data were collected, exploratory factor analysis (EFA) and confirmatory factor analysis (CFA) were conducted.

### Item generation

Through an extensive literature review, the team integrated items on BC myths from both English and Turkish articles. To identify culture-specific myths, in-depth individual interviews were conducted with 12 women aged 18 years and older from diverse age groups and education levels using a semi-structured questionnaire. The six questions addressed knowledge, risk perceptions, protective behaviors, and attitudes toward BC treatment. The interviews revealed myths widely believed by the public, some scientifically unvalidated and others supported by the literature. After refining the responses, 74 items were created from the literature review and interviews.

### Content analysis

The Content Validity Index (CVI) was calculated using the Lawshe technique, which is widely applied to assess content validity. Developed by Lawshe, this method evaluates expert consensus on each item^3^. It involves collecting feedback from experts to assess the reliability of items in a survey or measuring instrument. In this study, 17 experts (1 language, 4 scales, and 10 subject experts) evaluated the items. Items with CVI values below 0.59 were removed, based on the Lawshe table.

### Participants

Participants were recruited from three family health centers in Istanbul, Turkey, between April and June 2023. Sample size recommendations suggest at least 5–10 times the number of items^4-7^. With 50 items, the target was 400 participants. Eligible women were aged 18 years and older, Turkish-speaking, and willing to participate. A total of 406 women met the inclusion criteria.

### Ethical considerations

This study was performed following the Helsinki Declaration and has been approved by the Koc University's Ethics Committee (approval number: 2022. 419.IRB3.197). All participants’ written consents were obtained.

### Procedure

Women who met the inclusion criteria and accepted the study were informed about the purpose and method of the study. Women who volunteered to participate in the research were asked to sign the informed consent form. Then, volunteer women were asked to fill out the Introductory Information Form and the Breast Cancer Myths Scale (BCMS) (scale draft), prepared in line with the literature. Researchers met face to face with illiterate women and helped them fill out the forms. For the response style of the items in the item pool, a 5-point Likert-type scale scoring system was used to evaluate each item. Applicable for each item are 5 points (strongly agree), 4 points (agree), 3 points (undecided), 2 points (disagree), and 1 point (strongly disagree). The scale was conducted on 30 women again for a re-test 2 weeks after the initial test.

### Data analysis

The data analysis was performed using SPSS 25.0 and AMOS 24.0. Demographic variables were described with frequencies, means, and standard deviations. Item–total correlation and independent-sample t-test were used to evaluate internal validity, comparing the top and bottom 27% groups. EFA was done with principal component analysis (PCA) and varimax rotation^8^. The Kaiser-Meyer-Olkin (KMO) measure and Bartlett's test were used to assess data suitability for factor analysis. Items with a factor loading below 0.30 or loading highly on multiple factors were excluded^9^. Factors with an eigenvalue of 1.0 or more were retained. CFA used fit indices, such as chi-square, CMIN/DF (Minimum Discrepancy Function by Degrees of Freedom divided), GFI (Goodness of fit index), RMR (root mean square residual), SRMR (standardized root mean square residual), RMSEA (root mean square error of approximation), TLI (Tucker-Lewis index), CFI (comparative fit index). Internal consistency was evaluated with Cronbach's alpha (≥0.7) and test–retest reliability with the intraclass correlation coefficient (ICC)^10^.

## RESULTS

### Participants

A total of 406 participants completed the survey. Notably, 39.4% of the participants were at a higher education level, 24.9% were at a high school level, and 23.2% were at a primary school level. While 91.6% of them did not have any breast disease, 26.4% of their relatives had BC. While 40.4% of the participants practiced early diagnosis, only 21.2% stated that they went to a doctor for BC.

### Content validity

In this study, first, in line with the evaluations of the experts, the content validity ratio "([number of experts who say the item is necessary/appropriate]/total number of experts who expressed their opinions about the item)-1" was calculated for each item. Then, considering the "Lawshe Content Validity Criterion Values Table," the minimum value for the evaluation of 11 experts was determined as "0.59." Twenty-three items with CVI values less than or equal to 0.59 were removed. When the item-based CVI values varied between 0.60 and 1.00, the scale-based CVI was calculated to be 0.92.

To test the suitability and understandability of the scale items, a preliminary application was made to 12 women randomly selected from the society. In the preliminary application, only one item that caused incomprehensibility was detected, and the relevant item was removed. The scale, whose number of items decreased to 50, was made ready for validity and reliability study.

### Item reduction

The two items with the item–total correlation coefficient of ≤0.30 and causing the Cronbach alpha coefficient of the scale total to increase when deleted were removed. The item–total correlation coefficients of the remaining 48 items ranged from 0.31 to 0.66.

### Exploratory factor analysis

**Figure 1:**
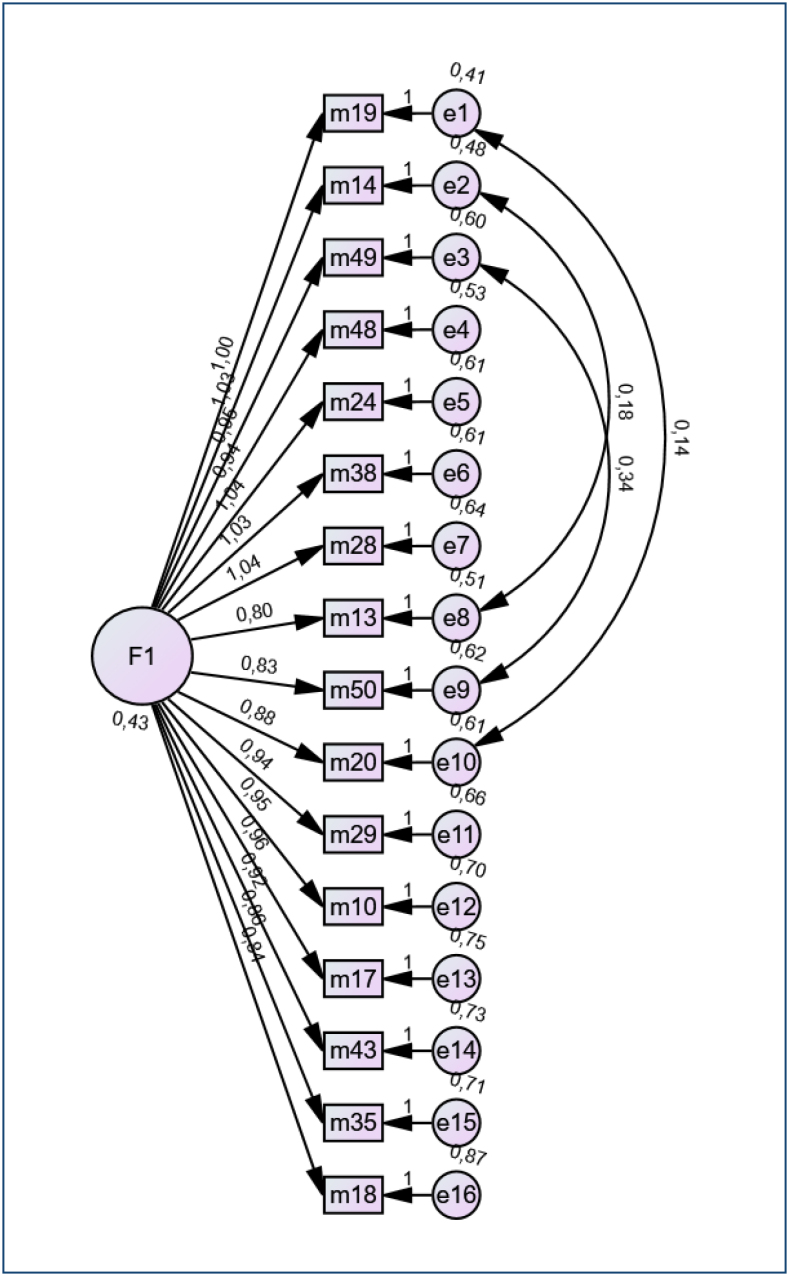
Path diagram.

In this study, the KMO coefficient (0.92) and Bartlett sphericity test (χ^2^=9,043.266, df=1,225, p<0.001) results supported that the data were suitable for factor analysis. The communalities of items were below 0.30, suggesting that they detracted from the factorability of the scale, and 32 items were removed. The factor analysis revealed the single-factor nature of the BCMS (Figure 1). As a result, 16 items remained. The extracted single factors explained a total of 42.2% of the total variance.

### Confirmatory factor analysis

The 16-item BCMS was tested with CFA. Modification indices were evaluated to improve the model fit. A high correlation was found between the remnants of items 13 and 14, items 49 and 50, and items 19 and 20. Covariance connections were added between the error term pairs of these items, and the model was rearranged. Considering the content of the items, adding these links was appropriate in terms of theoretical/logical sense. It was seen that the fit indices of the final model improved: χ^2^=313.803 (df=102, p<0.001), χ^2^/df=3.107, GFI=0.906, incremental fit index [IFI]=0.919, TLI=0.903, CFI=0.919, RMSEA=0.072 (90%CI 0.06–0.08, p<0.001), RMR=0.053, and SRMR=0.0515. All factor loadings were statistically significant and varied between 0.55 and 0.74. Factor loadings of the 16 items are presented in Table 1. Convergent validity, also known as aggregate validity, might be tested by calculating the average variance extracted (AVE) and construct reliability (CR) values. The AVE value of this model was 0.60 (>0.5), and the CR value was 0.92 (>0.7). Both the AVE and CR values provided evidence of the convergent validity of the final scale.

**Table 1:** Factor loadings of items (16 items).

Items	Factor loadings
Item 10: Breast cancer occurs only in women aged 45–64 years.	0.618
Item 13: Having a mammogram (breast film) for control causes breast cancer.	0.647
Item 14: For people diagnosed with breast cancer, having a mammogram (breast film) causes the cancer to spread.	0.730
Item 17: Finding a lump in the breast means breast cancer.	0.616
Item 18: Birth control pills cause breast cancer.	0.553
Item 19: Women with small breasts do not get breast cancer.	0.740
Item 20: Breast cancer does not develop in women who give birth.	0.637
Item 24: Biopsy (taking a sample from the mass) performed for the diagnosis of breast cancer causes the cancer to spread throughout the body.	0.676
Item 28: In breast cancer, medicinal plants are more beneficial than treatment with medication or surgery.	0.665
Item 29: If there is no mass in the breast, there is no need to go to the doctor.	0.620
Item 35: Surgery is required to diagnose breast cancer.	0.578
Item 38: Breast cancer does not occur before menopause.	0.671
Item 43: Having laser epilation under the armpit causes breast cancer.	0.616
Item 48: Receiving radiation therapy (radiotherapy) causes breast cancer.	0.679
Item 49: Dyeing hair after chemotherapy (treatment of cancer with medication) causes breast cancer to recur.	0.689
Item 50: Hair dyeing in women causes breast cancer.	0.641

### Reliability analysis

The reliability coefficient of the entire scale was found to be Cronbach's alpha=0.90. These values can be interpreted as an indicator that the scale is reliable. To determine the item discrimination of the items in the scale, the mean scores of the items were determined and item analysis was performed on the low 27% group and high 27% group. The difference between the mean group scores was analyzed using the independent-group test. There was a statistically significant difference between the upper and lower groups of 27% for all items in the scale, and it was seen that t-values were statistically significant (p<0.001). The test–retest reliability was conducted around 2 weeks after the initial test. In this study, ICC analysis was conducted to identify the test–retest reliability. These assessments determined that the test–retest reliability was excellent. The ICC was 0.946. The final scale version demonstrated excellent internal consistency (Cronbach's alpha=0.908) as a result of good levels of item-total correlation among almost all items (Table 2).

**Table 2:** Item–total correlations.

Items	Mean	Item–total correlations	Cronbach's alpha if the item is deleted
1	1.8	0.557	0.903
2	1.8	0.584	0.902
3	1.9	0.669	0.899
4	2.1	0.560	0.903
5	2.4	0.492	0.905
6	1.6	0.687	0.899
7	1.8	0.575	0.902
8	1.9	0.612	0.901
9	1.9	0.603	0.901
10	1.7	0.558	0.902
11	1.9	0.516	0.904
12	1.8	0.608	0.901
13	2.3	0.554	0.903
14	2.2	0.615	0.901
15	2.1	0.629	0.900
16	1.9	0.573	0.902

## DISCUSSION

This study aimed to develop a reliable, valid, and cost-effective instrument to assess BC myths in women. The BCMS consists of 16 items. Misinformation about BC can have serious consequences, affecting individual health and public health efforts. Addressing these myths is crucial for promoting accurate understanding, early detection, and effective management^11,12^. A valid, reliable scale helps nurses assess myth-related misconceptions, guiding health prevention and promotion efforts^13^. This study provides evidence of the BCMS's validity and reliability, with items derived from both literature review and interviews to reflect women's perspectives in Türkiye. Factor analysis is conducted to assess the possibility of grouping the items within a scale into distinct factors^14^. In the scales, it is enough to have a total variance explained by the factors of greater than 30%. The items with values between 0.55 and 0.74 are considered to have a moderate and high factor loading and values of 0.60 and above are considered to have a high factor loading^15^. According to the EFA performed, the scale had a single factor and explained 42.2% of the total variance. In CFA, the results are required to be examined via fit indices^16^. A χ²/df ratio below 5 is acceptable^17^, and our study's value of 3.107 indicated an acceptable fit. The RMSEA value of 0.072 was within the acceptable range^16^.

The fit indices range from 0 to 1, with values above 0.95 indicating a good fit and values above 0.90 indicating an acceptable fit^17^. In this study, the GFI, NFI, IFI, and CFI showed a good fit. For CFA, factor loadings are expected to exceed 0.30^18^. The absence of any increase in Cronbach's alpha when an item is deleted indicates no need to remove any item^19^. According to the literature, a Cronbach alpha coefficient below 0.40 indicates low reliability, 0.40–0.59 indicates moderate reliability, 0.60–0.79 is quite reliable, and 0.80–1.00 is highly reliable^20^. In this study, the Cronbach alpha was 0.90, indicating high reliability. The correlation coefficient for test–retest reliability was 0.91, showing consistent reliability and time invariance^21^. CR could be determined once CFA has demonstrated construct validity. CR is determined using a factor-loading approach^22^. Gefen et al^23^. recommend that CR coefficients greater than 0.70 are appropriate. The current study indicated that the single-factor BCMS has adequate convergent validity as supported by the higher levels of AVE and CR.

Rivas et al.^24^ determined that sociodemographic factors, such as race, religion, and especially education level, underscore the barriers that impact vulnerable groups’ access to screening programs. The findings of our study align with the conclusion that education plays a pivotal role in developing BC risk awareness and promoting early detection. BCMS provides a reliable tool to identify and address specific misconceptions that may serve as barriers to healthcare-seeking behaviors, particularly among vulnerable groups with lower education levels.

In clinical settings, by pinpointing specific misconceptions, healthcare professionals can tailor their educational interventions to address these inaccuracies, thereby empowering women with evidence-based knowledge about BC prevention, screening, and treatment. In educational settings, the BCMS can be integrated into public health campaigns, community education programs, and school curricula to evaluate and improve health literacy related to BC. For instance, the scale can be utilized to measure baseline knowledge before and after educational sessions, enabling educators to assess the effectiveness of their programs and refine their content to better address the needs of their audience.

The BCMS can facilitate the exploration of correlations between myth prevalence and demographic, cultural, or psychological factors, thereby informing targeted interventions.

### Limitations

The study aimed to reach individuals with different education levels in primary healthcare institutions in Turkiye. However, the small number of primary school graduate participants may affect the representativeness of the sample. This could have a potential impact on the generalization of the study and limit the applicability of the results to other groups.

## CONCLUSION

The BCMS is a highly effective tool for a detailed assessment of misinformation and beliefs about BC. The use of this measurement tool can increase the effectiveness of BC awareness and education efforts and encourage society to access accurate information. Additionally, translating the BCMS into clinical applications may be an important step in identifying misinformation about BC and designing more effective educational programs. This may present an opportunity to provide better patient care by guiding healthcare professionals to provide accurate information and correct false beliefs. In this way, the role of BCMS in clinical practice can be considered important to increase access to accurate information about BC and develop effective interventions.
